# Mental disorders among sexual and gender minorities

**DOI:** 10.1192/j.eurpsy.2022.2286

**Published:** 2022-09-01

**Authors:** S. Khouadja, F. Zaouali, M. El Echi, C. Najjar, L. Zarrouk

**Affiliations:** University Hospital of Mahdia, Tunisia, Psychiatry, Mahdia, Tunisia

**Keywords:** Sexual Minorities, Mental Disorders, Bisexuality, Homosexuality

## Abstract

**Introduction:**

The term “Sexual and Gender Minorities” includes lesbian, gay, bisexual, transgender, queer, intersex and/or asexual populations. It was introduced in the MeSH Database in 2018. Mental health research on sexual and gender minority populations is gaining momentum.

**Objectives:**

To describe mental disorders among sexual and gender minorities.

**Methods:**

This is a review of the literature via Medline. The database was searched using the keyword combination “sexual gender minorities” OR “homosexuality” OR “bisexuality” OR “transgender persons” OR “intersex persons” AND “mental disorders”. The filters applied were Full text, Meta-Analysis, Systematic Review and in the last 5 years.

**Results:**

A total of 59 articles were included. The lowest rates of depression and anxiety were reported among heterosexual people. Depressive symptoms, suicidality, interpersonal trauma exposure, substance use disorders, anxiety, and general distress have been consistently elevated among transgender and gender non-conforming people. Among transgender people, the prevalence of binge drinking ranged from 7%-61%. Depression was the most frequent mental disorder among sexual minority men (43.2%) followed by anxiety (32.2%), suicidal ideation (21.2%), suicide plans (6.2%) and suicide attempts (7.3%). Eating disorders were more frequent among sexual minority women compared with heterosexual peers. Compared with heterosexual youth, sexual minority youth had 123% to 623% higher odds of lifetime substance use, 82% to 317% higher odds of depressive symptoms and suicidality and 20% to 280% higher odds of violence victimization.

**Conclusions:**

The prevalence of mental disorders is high among sexual and gender minorities for whom mental health prevention and treatment programs are needed.

**Disclosure:**

No significant relationships.

